# The Evolving Landscape of Exosomes in Neurodegenerative Diseases: Exosomes Characteristics and a Promising Role in Early Diagnosis

**DOI:** 10.3390/ijms22010440

**Published:** 2021-01-04

**Authors:** Simran Rastogi, Vaibhav Sharma, Prahalad Singh Bharti, Komal Rani, Gyan P. Modi, Fredrik Nikolajeff, Saroj Kumar

**Affiliations:** 1 Department of Biophysics, All India Institute of Medical Sciences, New Delhi 110029, India; sr_1997du@yahoo.com (S.R.); vaibhavsharma.aiims@gmail.com (V.S.); bharti.pbh@gmail.com (P.S.B.); 2 Department of Biotechnology, Amity University, Mumbai 410206, India; komal.pims89@gmail.com; 3 Department of Pharmaceutical Engineering and Technology, Indian Institute of Technology, Banaras Hindu University, Varanasi 221005, India; gpmodi.phe@iitbhu.ac.in; 4 Department of Health Science, Lulea Technical University, 97187 Lulea, Sweden

**Keywords:** salivary exosomes, neurodegenerative diseases, biomarkers, Alzheimer’s disease, Parkinson’s disease

## Abstract

Neurodegenerative diseases (ND) remains to be one of the biggest burdens on healthcare systems and serves as a leading cause of disability and death. Alzheimer’s disease (AD) is among the most common of such disorders, followed by Parkinson’s disease (PD). The basic molecular details of disease initiation and pathology are still under research. Only recently, the role of exosomes has been linked to the initiation and progression of these neurodegenerative diseases. Exosomes are small bilipid layer enclosed extracellular vesicles, which were once considered as a cellular waste and functionless. These nano-vesicles of 30–150 nm in diameter carry specific proteins, lipids, functional mRNAs, and high amounts of non-coding RNAs (miRNAs, lncRNAs, and circRNAs). As the exosomes content is known to vary as per their originating and recipient cells, these vesicles can be utilized as a diagnostic biomarker for early disease detection. Here we review exosomes, their biogenesis, composition, and role in neurodegenerative diseases. We have also provided details for their characterization through an array of available techniques. Their updated role in neurodegenerative disease pathology is also discussed. Finally, we have shed light on a novel field of salivary exosomes as a potential candidate for early diagnosis in neurodegenerative diseases and compared the biomarkers of salivary exosomes with other blood/cerebrospinal fluid (CSF) based exosomes within these neurological ailments.

## 1. Introduction

There has been an ever-increasing interest in the development of early diagnostics and novel treatment modalities for neurodegenerative diseases. These nervous system-related degenerative diseases have highly burdened the population worldwide due to their associated mortality and morbidity. The irreversible damage, which occurs in neurodegeneration, together with the concomitant increase in the rate of progression throughout the treatment has largely impeded the countermeasures. The neurodegenerative disorders encompass various diseases that include Alzheimer’s disease (AD), Parkinson’s disease (PD), Huntington’s disease (HD), and amyotrophic lateral sclerosis (ALS). AD and PD remain the first and second most common neurodegenerative diseases worldwide [1]. The major cause is the aggregation of misfolded proteins, which leads neuronal cells to degenerate in various regions of the brain as per the pathologic and the physiological changes caused by each disease. 

The search for efficient biomarkers of these diseases is a vividly explored field nowadays. Till now, extensive studies on biofluids based diagnostics are available in the literature [2,3,4,5,6]. Proteomic studies from complete blood, cerebrospinal fluid (CSF), saliva, and urine are also reported. Each of these biofluids-based methods suffers from a variety of limiting factors, for instance brain-derived biomarkers are typically present at moderately low concentrations in the blood due to the blood-brain barrier preventing the free passage of molecules between the central nervous system (CNS) and blood compartments. Additionally, most of the biomarkers related to neurodegenerative pathologies are also expressed in non-cerebral tissues, which may confound their measurement in the blood. Furthermore, the analyte of interest may undergo proteolytic degradation by various proteases in plasma. CSF collection is considered a highly invasive procedure and suffers from disinclination from both patients and clinicians [7].

Recent advances in nuclear magnetic resonance (NMR) and mass spectrometry resulted in a rapidly emerging field of metabolomics-based biomarker development. Metabolites like glycerophosphocholine and d-glucosamide were reportedly present in AD patients in comparison to normal healthy controls [8]. Another study consisting of mild cognitive impairment (MCI) patients who did and did not progress to AD within 2 years’ time frame has shown the progression to AD was correlated with the expression of 2,4-dihydro butanoic acid [9]. Additionally, metabolites like β-alanine, aspargine, l-cysteine, l -methionine, l-arginine, lysine biosynthesis, and metabolism were recorded to be significantly different among controls and AD subjects [10]. 

**Figure 1:**
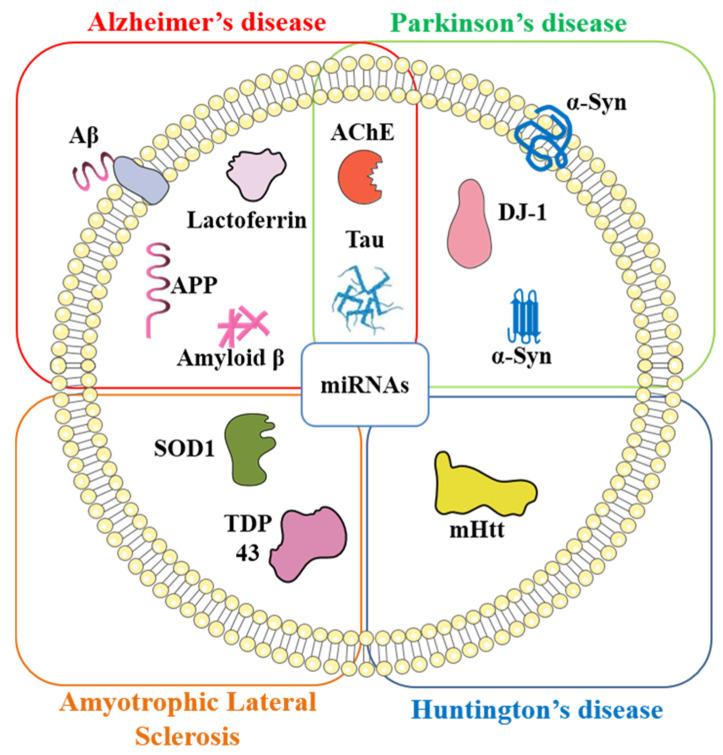
Schematic representation of key exosomal biomarkers in neurodegenerative diseases. Amyloid-beta (Aβ), amyloid precursor protein (APP), acetylcholinesterase (AChE), alpha-synuclein (α-syn), super-oxide dismutase 1 (SOD1), tar-DNA binding protein-43 (TDP-43), mutant huntingtin protein (mHtt), and micro RNAs (miRNAs).

Exosomes based biomarker detection is a novel and rapidly evolving field within neurodegenerative disease diagnostics (Figure 1). The exosomes are single lipid bilayer membrane-enclosed vesicles with a diameter of 30–150 nm, known to be found in intraluminal/extracellular spaces as observed by electron microscopy, and are secreted by all types of cells. Exosomes are known to be present in plasma, serum, urine, CSF, saliva, breast milk, and other secretions [11]. The role of exosomal protein cargo and their content modifications with the varying disease pathology has made them a promising biomarker candidate [12]. They are known to be involved in cell–cell communication [13] and; aid in the regulation of synaptic plasticity and nerve regeneration process [14]. These processes are through various exosomes mediated signaling cross-talks [15,16,17,18,19]. The generalized mechanism of transmission of signals in the CNS is through a chemical and electrical synapse. Additionally, in CNS, the exosome release is regulated by the glutaminergic neurons, when Ca2+ enters through N-methyl-D-Aspartate and hydroxyl-5-methyl-4-isoxazole propionic acid (AMPA) [20]. Furthermore, exosomes are also known to spread the infectious prion protein molecules, hence are responsible for the seeding of infectious molecules [21]. 

The oligodendrocyte-derived exosome cargo plays a key role in myelin sheath biogenesis as well as having a prime role in tissue regeneration [22,23]. Exosomes are majorly responsible for carrying myelin proteolipid protein (PLP) which is in turn responsible for myelin sheath formation, this phenomenon was also confirmed in vitro, by a culture of oligodendrocyte in presence of calcium-ionophore ionomycin [24]. In general, exosomes can cross the blood–brain barrier, which comprises of the brain macrovascular endothelial cells, and tight junctions. Therefore, exosomes are thought to play an important role as a drug delivery vehicle also [25,26,27,28]. 

In this primer, we provide a broader description of exosomes, their isolation, and characterization methodologies in general as well as from a neuropathologic perspective. The role of exosomes in neurodegenerative pathologies along with their most updated exosomal markers is also discussed. Finally, our current understanding of a relatively newer area of salivary exosomes as a potent biomarker for neurodegenerative diseases is elaborated.

## 2. Exosomes: Biogenesis, Composition, and Their Diverse Functions

There exists a well-established process through which a variety of cells release different hormones or neurotransmitters, likewise, most cells have an evolutionarily conserved mechanism to secrete a myriad of membranous vesicles that are known as extracellular vesicles (EVs) [29]. These EVs are thought to be involved in clearing cellular junk, however, in recent years, the focus has been shifted to look into their auxiliary functions [30,31]. EVs are also involved in cell-to-cell communications by an exchange of different biomolecules including nucleic acids, lipids, and proteins, and thus held responsible for maintaining cellular homeostasis and in most cases results in the progression of current pathological manifestations [32]. Generally, EVs are highly heterogeneous in nature due to the associated cargo which is dependent on their releasing cell type. There are subtypes of extracellular vesicles based on their biogenesis and size, exosomes, and microvesicles [29,33]. Though there are some overlapping characteristics of both of them, the primary differences between exosomes and microvesicles are shown in Table 1. 

**Figure 2:**
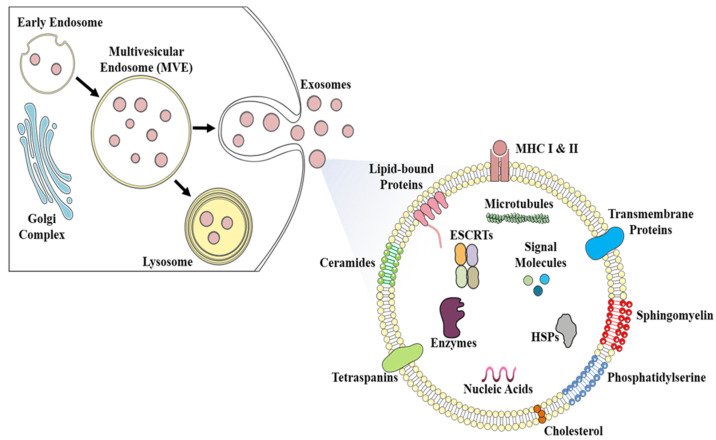
Illustration showing key events in the process of exosome biogenesis including the compositional details of a mature exosome.

Exosomes were first described in 1981 [34] and were initially investigated by Johnstone et al. [35]. They noted the secretion of small vesicles (30–150 nm in diameter) by reticulocytes in sheep, explained their endocytic origin, and coined the term “exosomes” for these extracellular vesicles. The biogenesis of exosomes has been studied with several biochemical approaches in addition to transmission and immuno-electron microscopic (TEM/IEM) methods [36,37,38] (Figure 2). Briefly, multivesicular endosomes (MVEs) contain intraluminal vesicles (ILVs), which are formed by inward budding of the endosomal system. These ILVs are responsible for secreting exosomes after fusing with the plasma membrane [39,40,41]. The biogenesis and release of exosomes are different from microvesicle formation because microvesicles are formed by the budding of the plasma membrane whereas exosomes are released by the fusion of MVEs to the plasma membrane [29,42]. It has been found that the biogenesis and subsequent release of exosomes are fairly dependent on the physiological state and conditions of both the originating cells as well as target cells, therefore, different exosomes have varying protein, lipid, and nucleic acid profiles [29]. The biogenesis of exosomes is initiated when cargoes are secreted by Golgi bodies and transported to targeted endosomal membranes which are subsequently matured into MVEs with an average diameter between 250–1000 nm. Several ILVs (30–150 nm) are formed in the lumen of MVEs during its maturation process by inward budding of the endosomal membrane of MVEs [40]. During this invagination, designated cargoes are sorted and segregated, and are further incorporated into the forming ILVs. The highly selective cargo segregation and sorting require specialized sorting pathways including the endosomal sorting complex required for transport (ESCRT)-dependent, and ESCRT-independent pathways [43,44,45]. ESCRT is an evolutionarily conserved protein complexes family, having ESCRT-0, ESCRT-I, ESCRT-II, and ESCRT-III proteins, which involves in cargo sorting and membrane forming of MVEs and ILVs [45]. The ESCRT-0 and ESCRT-I proteins segregate and associate ubiquitylated cargoes to lipid microdomains of the membrane of MVEs, followed by invagination and formation of MVEs and ILVs by ESCRT-II and ESCRT-III protein complexes. Some other proteins are also involved in the ESCRT machinery which includes ALG-2 interacting protein X (ALIX), tumor susceptibility gene 101 (TSG101), and vacuolar protein sorting-associated protein (VPS4). In the ESCRT independent mechanism, tetraspanins (CD63, CD81, CD82, CD37, and CD9) and chaperones (HSP60, HSP70, and HSP90) play a major role by clustering cargoes to lipid microdomains and successive formation of MVEs and ILVs [39,41,46]. Tetraspanins CD63 and CD81 are highly enriched on the membranes of ILVs and thus regarded as housekeeping markers for exosomes [46,47]. Syntenin is also involved in the ESCRT mechanism and has a role in the recycling and sorting of cargoes. It has been reported that both ESCRT dependent and independent mechanisms work in the biogenesis of exosomes, however, the selection of mechanism is highly dependent on their respective cargoes and cell types [41]. Energetically, the transfer and docking of newly formed MVEs with the plasma membrane are dependent on Ras associated binding (Rab) family of GTPases and the fusion of the MVEs to the plasma membrane requires soluble NSF-attachment protein receptor (SNARE) protein complexes [43,48,49]. After fusion to the plasma membrane, MVEs release the ILVs, which are termed exosomes upon release from the cell to the extracellular regions. Not all matured MVEs are transferred to plasma membranes, some of them are also directed to the lysosomal pathway for degradation [43,48].

The composition of exosomes is diverse and may reflect its origin cells or tissues. Their content includes proteins, lipids, enzymes, and nucleic acids, which play an important role in cell to cell communications and are responsible for delivering various signal molecules to both proximal and distant locations. Some of the most common cargoes on exosomes are transmembrane proteins, cytoskeletal proteins, and heat shock proteins; various lipids; and various types of RNAs e.g., mRNA, microRNA (miRNA), non-coding RNA (ncRNA), mitochondrial DNA (mtDNA), and single-stranded and double-stranded DNAs (ssDNA and dsDNA) [29,32]. Some comprehensive databases of exosomes like ExoCarta [50], Vesiclepedia [51], and EVpedia [52] are also available which have detailed information on exosomal components including proteins, lipids, and nucleic acids as well as included different methodologies of exosomes isolation and characterization. Exosomes contain certain conserved proteins that include proteins involved in ESCRT dependent exosomal biogenesis such as ALIX and TSG101, and ESCRT independent tetraspanin family of proteins like CD63, CD9, CD37, CD81, and CD82 [29,42,53]. Since these proteins are absent on other types of vesicles, these can be considered as “hallmark exosomal markers”. Tetraspanins are categorized into a class of transmembrane proteins that interact with other proteins like integrins and thereby result in the transport and fusion of exosomes and helps to establish a connection with target cells [43,46,47]. Additionally, Rab GTPases, annexins, and flotillin assist in the efficient transport and fusion of exosomes [54,55]. Another important protein, syntenin, is involved in the clustering of exosomal proteins to transmembrane domains, especially CD63. Higher expression of syntenin is correlated with CD63 enrichment onto the surface of exosomes [46,47]. 

The exosomes are generously enriched in lipids, especially lipid rafts such as ceramides, sphingomyelin, cholesterol, sphingolipids, glycerophospholipids, and glycosphingolipids. The exosomal membrane has lysophosphatidic acid which plays an important role in the formation of ILVs from MVEs. Some lipids are found in lesser quantities which include phosphatidylserines, phosphatidylcholine, and phosphatidylinositols. Exosomes also have prostaglandins and some enzymes such as phosphatases, glycosidases, lipases, and proteases. The enzymes found in exosomes generally represent exosomal origin cell types and their metabolic activity [11,56]. 

The exosomes also contain nucleic acids that play an important role in intracellular communications as well as in the pathophysiology of a variety of diseases. Both RNA and DNA are found as exosomal cargo and their concentration and composition are dependent on the origin and the target cells [57,58,59]. Exosomal miRNAs play a significant role in intracellular communications and gene regulation. The miRNAs have been discussed in several studies due to their potency as a diagnostic marker and in the understanding of disease progression and pathology [60]. Michael et al. [60] successfully showed the isolation and characterization of salivary exosomal miRNAs (miR-31, miR-17-92, miR-125a, and miR-200a) in an easy and non-invasive way. Some studies also reported mtDNA, ssDNA and dsDNA, and genomic DNA in exosomes from various sources, however, their detailed roles and sorting mechanism are still a topic of active research and most details are yet to be elucidated [57,58].

## 3. Ultrastructural, Biochemical, and Size Based Characterization of Exosomes

The characterization of exosomes can be done through physical and biochemical principles. The properties such as size, concentration, and ultrastructural morphology. can be exploited for physical analyses whereas the content and composition of exosomes (protein, nucleic acids, lipids, and enzymes) can be studied using biochemical analyses. Physical techniques commonly practiced are nanoparticle tracking analysis (NTA), dynamic light scattering (DLS), electron microscopy, and atomic force microscopy, whereas the biochemical techniques include Western blotting, ELISA, qRT-PCR and flow-cytometry [61,62,63]. Here, we have summarized some of the most common techniques for the characterization of exosomes.

### 3.1. Physical Analysis of Exosomes

Electron microscopy: Electron microscopy (EM) utilizes a focused beam of accelerated electrons, instead of light, under a vacuum that interacts with the specimen to produces images with superior resolution than that of light microscopy [64]. Scanning electron microscopy (SEM) can be used to scan the surface topography and can also aid in precise quantification of elements present within the samples. EM can, furthermore, be used on thin sections of the specimen to study their ultrastructural morphology by transmission electron microscopy (TEM). Both techniques are well established and being used to study the exosomes, moreover, TEM is considered as a gold standard technique to characterize exosomes. A variation of EM can also be used to label different surface markers by immunogold-antibodies (immuno-EM). Under EM, exosomes display cup-shaped morphology due to fixation, dehydration, and staining of the samples [65]. The main advantage of studying exosomes through EM includes the production of images at a sub-nanometer level with surface topography, ultrastructural morphology, and labeling of marker proteins effectively. However, the disadvantages are a cumbersome and laborious process that requires expensive technical setup. Furthermore, the optimization of sample processing must be done to obtain good results which can be a tedious task because improper fixation and dehydration can lead to artifacts [64,65]. Cryo-electron microscopy (cryo-EM) is a modification of EM where samples are analyzed at extremely low temperatures using liquid nitrogen [66]. This method is advantageous to EM because fixation, dehydration, and staining are not required and since the samples are frozen, less processing is needed which minimizes the changes in morphology [66,67,68], resulting in more accurate and detailed images. The exosomes display proper round morphology in cryo-EM, demonstrating that the cup-shaped morphology of EM analyzed exosomes is the result of fixation and dehydration of the samples.

Atomic force microscopy (AFM): AFM is a type of scanning probe microscopy, based on Hooke’s law, which uses a small mechanical cantilever that scans the surface of the sample resulting in details of surface topography and presence of substructures [69]. There are two types of information recorded by AFM, amplitude-modulating, and phase-modulating; the amplitude modulation provides information on surface topography, and the phase-modulating records information of substructures [69,70]. The advantages of AFM in the characterization of exosomes are higher resolution images as well as detailed sub-structural information. It shows a circular morphology of exosomes and phase images display substructures like lipids and proteins. Moreover, fewer stages of sample preparations are needed to view exosomes under AFM. However low output, cumbersome quantifications, and expensive instrument setup are drawbacks of a typical AFM setup [69,70].

Nanoparticle tracking analysis: NTA is a particle tracking method that measures the size (hydrodynamic diameter) and concentration of particles in a suspension based on the Brownian motion of the particles [71,72]. The laser is used to illuminate the particles in suspension and the light scattered by particles is recorded by a camera that also documents the paths of each particle. This information is used to determine the size and concentration of the particles in suspension using the Stokes–Einstein equation [71,72]. NTA can be used to characterize particles such as exosomes by measuring size and concentration in a suspension simultaneously, also, exosomes can be detected by fluorescent tags that provide surface marker details, if coupled with suitable antibodies [72,73]. The detectable size range of particles is typically between 10 nm and 1 um, therefore proper optimization is needed for precise quantification of size and concentration in heterogeneous populations of exosomes [74].

Dynamic light scattering: DLS is similar to NTA as it is also based on Brownian motion and Stokes–Einstein equation however it provides information only based on intensity-based distribution therefore the fluctuation in intensity over time is used to determine the size of the particle [75]. DLS is a simple and fast technique but its use is limited to homogenous populations of exosomes due to its biasness towards the presence of larger vesicles, hence it cannot be used for heterogeneous populations of exosomes and generally suffers from having lower resolution than NTA [76,77].

Tunable resistive pulse sensing (TRPS): TRPS is based on the Coulter principle that measures transient changes in electric current, generated by particles while passing through a size-tunable pore in membrane to determine the size of the particles [78,79]. TRPS is a useful method for the characterization of exosomes as it can measure size, concentration, and zeta potential simultaneously [80]. However, TRPS can only be used to characterize smaller sized exosomes (<50 nm) and cannot distinguish between exosomes, protein aggregates, or other nanoparticles. Additionally, for better results, the instrument should be calibrated before each sample to confirm the accurate determination of exosome concentration [78,79,81].

### 3.2. Biochemical Analysis of Exosomes

Western blotting (WB) and enzyme-linked immunosorbent assay (ELISA) are conventional methods based on antigen–antibody reaction, and the most commonly used techniques to characterize exosomes to demonstrate the presence of targeted exosomal proteins [82]. In WB, isolated exosomes are subjected to lysis for total protein isolation followed by separation through sodium dodecyl sulfate-polyacrylamide gel electrophoresis (SDS−PAGE) and transfer of separated protein to a membrane for immunoblotting of targeted exosomal proteins. WB can provide information about the size and expression/abundance of different proteins but it is a time-consuming technique which requires a significant time for preparation and processing [83]. ELISA requires exosomal target-specific antibody immobilized to a solid surface that will capture the exosomal targets followed by labeling of a detection antibody [84,85]. High-throughput measurements can be done using ELISA because it is a less time-consuming technique than WB [86]. However, both the techniques suffer from some drawbacks such as low specificity and quality, and higher cost of consumables (antibodies). On the other hand, real-time quantitative reverse transcription polymerase chain reaction (qRT-PCR) is used to quantify the content of exosomes, such as different nucleic acids, specifically mRNAs and miRNAs. This technique is based on selective amplification of target nucleic acid sequence by polymerase chain reaction which can be detected in real-time using fluorescent probes [87]. This technique has several advantages such as the requirement of very low sample volume, higher sensitivity, high resolution, high-throughput analysis, as well as relative and absolute quantification [88,89]. This technique is also helpful in studying and characterizing the transcriptome of the given samples. However, the drawbacks of this technique are the total amount of RNA cannot be measured as well as the measurements of only known specific sequences can be made.

Flow cytometry (FCM) is a sensitive technique for characterizing small particles like exosomes by measuring scattered light or fluorescence activation. The samples, labeled with fluorescent tags or antibodies, are passed through the laser of different wavelengths producing scattered light in forward scatter (FSC) and side scatters (SCC) profiles. FSC denotes the size of the particles whereas SCC indicates internal structure details and is more sensitive than FSC [90,91]. For the characterization of exosomes, the lipophilic fluorescence tags or antibodies specific to exosomes can be used for both quantitative and qualitative analysis [92]. The advantages include the use of unprocessed samples, faster output, quantification, and reproducibility, however, the drawback is its low resolution (200 nm), and thus has limited sensitivity in characterizing exosomes [93,94]. To overcome this problem, in recent years many modifications have been done to FCM, such as the development of nano-FCM which has a higher resolution limit (40 nm) [95,96]. Moreover, being a powerful, sensitive, and sophisticated technique, accurate optimization, and standardization are needed for better result output.

Briefly, we have tabulated the general techniques used to characterize exosomes along with their pros and cons in Table 2.

## 4. Exosomal Biomarkers and Their Role in Neurodegenerative Diseases

### 4.1. Alzheimer’s Disease

**Figure 3:**
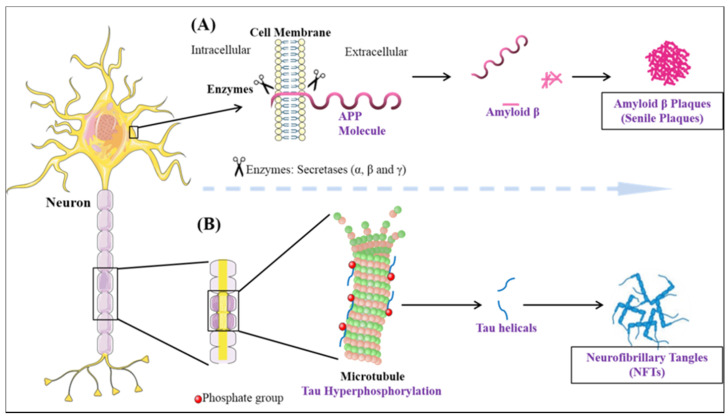
The neuropathological hallmarks of Alzheimer’s disease: (**A**) formation of amyloid-beta plaques (**B**) formation of neurofibrillary tangles.

Dementia is an umbrella term that is broadly used for the loss of cognitive functioning and memory. Medically, it can be referred to as chronic brain dysfunction. According to the recent statistical reports, the prevalence of dementia is thought to increase from 3% (age group 70–75 years) to 20–25% among those with the age approaching 85 years. It is predicted, the number of individuals currently suffering will be doubled every 20 years and may account for 81.1 million diseased people by 2040. The developing countries are among the highest sufferers (60% of all the global dementia cases in 2001, expected to rise to 71% by 2040) [97]. Although, in aged individuals, memory loss is not uncommon, the effect on one or more domains of cognition within the brain resulting in an altered social behavior is considered as a major characteristic of dementia [98]. The majority of dementia cases are dictated by AD pathology. Two-third of individuals suffering from dementia as observed in population studies have Alzheimer’s disease [99]. Therefore, on the whole, AD accounts for 70% of dementia cases [100]. The histopathological changes occurring in AD brain can be divided into two processes: Firstly, the formation of extracellular plaques (senile plaques) by deposition of amyloid-beta and secondly, the intercellular tangles/neurofibrillary tangles (NFT’s) originating from paired helical filament (PHF) of microtubule-associated protein tau [101] (Figure 3). In a normal aging brain, the presence of plaques is reported, but the neurotoxic amyloid-beta (Aβ) species forming plaques are responsible for the disease pathology [102]. The aggregates of amyloid-beta found extracellularly are formed due to the deteriorative defect in the amyloid precursor protein (APP) machinery [103]. In the most common cases, the proteolytic cleavage of the transmembrane glycoprotein APP by the help of β, γ (presenilin-1, presenilin-2) secretases aids in the formation of various Aβ species [104,105,106,107,108]. The most prominent reason for defective APP machinery that leads to aggregative nature Aβ is the defective substrate i.e., APP, or the presenilin enzyme (PSEN), which is sufficient to cause the disease [109]. The genetic mutation in the PSEN gene as well as the presence of apolipoprotein ε4 genotype makes an individual more prone to cognitive impairment and AD [110,111,112,113,114]. The formation of plaque is known to be caused by Aβ42, which is crowned as the culprit to cause disease and also portrays fibrillation inducing capabilities. Evidence suggests that polymerization of Aβ is a complex process linked with various metastable intermediaries and thus is termed as nucleated conformational conversion [115]. The elusive behavior of Aβ oligomer in the causation of AD is not very prominently known, but the present evidence suggests that the soluble oligomeric species derived from the brain strongly correlates with the decline in cognition better in comparison to plaques [116]. The soluble oligomer toxicity machinery works by three different molecular pathways [117]. Therefore, the perturbations in the maintenance of Aβ homeostasis in between the CNS and peripheral system lead to the accumulation of toxic species.

The role of exosomes in the cell to cell communication and pathology of AD is beginning to unfold [118]. The exosomal protein cargos containing APP, Aβ, and tau facilitates intercellular communication and leads to further propagation of Aβ and tau pathologies [119]. In the case of AD, the loss in function of the endosomal-lysosomal system due to a heterozygous or homozygous Apo E4 genotype is one of the prime reasons for an increased exosome production [120,121,122]. The proteasomal and lysosomal system dysfunction is common in AD and is the reason why the APP containing MVE’s fuses with the plasma membrane. Generally, the late endosome can either fuse with a lysosome, resulting in the digestion of their inherent material, in presence of hydrolases, or the fusion of MVE’s consisting of ILV’s with plasma membrane which results in the formation of exosome [123,124]. Exosome shows ambiguity in its role with its, either, neuroprotective or neurodegenerative nature. In AD, the extracellular vesicles have been proven to participate in the dispersing of Aβ and thereby exuberating Aβ pathology [125,126,127,128]. A similar exosomal dependent spread of hyperphosphorylated tau is also reported [129,130,131,132]. It is also observed that EVs contain APP, C-terminal fragments of APP, and the various isoforms of Aβ. 

The exosomal cargo content including proteins, lipids, and nucleic acids are gaining importance as prospective biomarkers. In the case of diseases like AD, where behavioral symptoms occur much later in life than the actual events of disease progression, the search for identification of early screening markers has been of utmost importance. Within CSF derived exosomes from severe AD patients, the decreased expression levels of Aβ were reported [133]. In another study from plasma-derived exosomes, Aβ was higher and their level was lower when compared to exosomes derived from other sources [134]. The level of soluble Aβ42 and other proteins involved in the Aβ42 generating pathway is higher within the astrocytic derived exosomes in comparison to the neuronal exosomes [135]. The ratio of p-tau/total tau also increases in the case of AD [136]. The activity of lactoferrin and acetylcholinesterase was also assessed in AD patients [137,138].

### 4.2. Parkinson’s Disease

**Figure 4:**
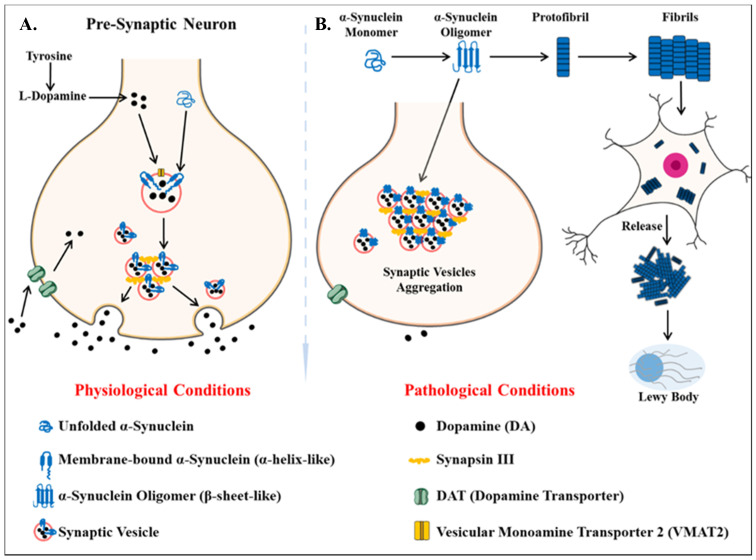
Key differentiation in the normal physiological state and pathological state of dopamine metabolism and homeostasis of alpha-synuclein protein (α -syn) (**A**) the normal physiological state: Homeostasis of alpha-synuclein protein maintained (**B**) the perturbations in alpha-synuclein homeostasis as well interneuronal aggregation of α-syn oligomer and formation of lewy bodies.

Parkinson’s disease is the second most common neurodegenerative disease after AD [139]. It is a movement disorder of older age with 2–3% of > 65 years being affected [140]. The incidence rate of PD ranges from 5 to > 35 newer cases per 100,000 individuals worldwide [141]. The onset of behavioral symptoms of PD is rare before 50 years of age [141,142]. The occurrence of this disease is thought to be more common in men than in women [143], with an exception of the study completed in Japan that concluded the prevalence is gender unbiased [144]. The men’s susceptibility to the disease has led to the notion that women’s sex hormones may portray a protective mechanism against this disease, however, this view is still debatable [145]. Parkinson’s disease is a prion-based neurodegenerative disorder [146], which is known to be associated with the loss of dopaminergic neurons in the substantia nigra pars compacta portion of the midbrain and the prime histopathological change i.e., lesions are seen within this region. Majorly the lesions are in the ventrolateral portion of substantia nigra [147,148]. The appearance of lesions is due to the depigmenting of the dopaminergic neurons [149]. The key molecular and neurophysiological mechanism of PD pathology is the intraneuronal aggregation of the misfolded alpha-synuclein protein and the presence of Lewy bodies (Figure 4). The presence of Lewy bodies is the prime neuropathological change accompanying aggregation of oligomeric alpha-synuclein (α-syn) [150]. α-syn acts as a molecular chaperone and has a role in intracellular trafficking and synaptic vesicle transportation [151,152]. α-syn is required for the release of neurotransmitters as it facilitates the association of synaptic vesicles with SNAREs assembly [153]. The other accomplice of PD pathology, the Lewy bodies, which spread from the serotonergic and cholinergic neurons to the limbic and the neocortex regions [154]. A Parkinson’s affected individual shows both motor and non-motor symptoms. The major motor symptoms are bradykinesia, resting tremor, postural instability, stooped structure, freezing, or rigidity. The most significant non-motor symptoms are hyposmia, rapid-eye ball movement sleep disorder (RBD), constipation, urinary dysfunction, depression, and hallucinations accompanied by impairment in cognition [155,156,157,158]. The spread of PD occurs in the Braak staging manner [159]. The symptoms of PD can be easily managed by the currently available treatments. The highly efficacious therapies which use the L-dopa and the deep brain stimulation have made PD the only neurodegenerative disease with manageable symptoms [160,161,162,163]. The mechanisms of aggregated α-syn neurotoxicity include mitochondrial defects, proteasomal effects, endoplasmic reticulum stress, and inflammatory responses [164]. The oligomeric α-syn results in a multitude of conditions in mitochondria and endoplasmic reticulum: Mitotoxicity by a decrement in calcium retention time and increasing cytochrome c release [165]. The transgenic mouse model with an A53T point mutation of the SNCA gene results in deleterious effects on the ER protein quality due to the overproduction of α-syn oligomers [166].

The genetically linked familial form of PD is associated with the point mutation in the SNCA gene, encoding the α-syn protein [167]. The perturbations in proteostasis and degradation of α-syn are cardinal to the development of PD pathology [164,168]. Generally, α-syn is present in the monomeric form, but after the acquisition of neurotoxic properties, it undergoes oligomerization and further aggregates to form protofibrils [169]. The degradation of α-syn occurs by the lysosomal autophagy system (LAS) and proteasomal pathway [168]. The LAS pathway has mainly been associated with the clearance of α-syn oligomeric assemblies [168,170]. 

The role of exosomes in PD results in the seeding of intraneuronal α-syn in a prion-like manner. Exosomal machinery serves as an auxiliary mechanism for dissipating early molecular changes of PD pathology to other cells [171]. The genetic mutations occurring in PARK-LRRK2′s gene locus 12q12 are linked with the faulty LAS machinery [172]. The G2019S mutation in LRRK2 is associated with the impaired functioning of the LAS pathway, thus corroborating with the disturbed homeostasis of α-syn leading to more aggregated form and further results in depigmentation of dopaminergic neurons. The α-syn containing MVB’s are formed from α-syn possessing endosomes and thus aid in the transmission of PD pathology after fusion with the plasma membrane [173,174]. It is also reported that the release of exosomal associated α-syn is regulated by the intracellular concentration of calcium [175]. The ambient environment of exosomes is also suggested to promote aggregation of α-syn and aid in spreading the PD pathology [176]. It was observed that α-syn increases the secretion of exosomes by microglia BV-2 cells of mice [177]. Moreover, the inhibition of the LAS pathway has shown an increased production of exosomal cargo with α-syn and decreased intra-neuronal α-syn aggregation, thus establishing the neuroprotective role of exosomes [175]. An experimental observation suggests that R1441C LRRK2 mutation aids in the induction of large MVB’s and thereby facilitating the exosome release.

In recent years, the search for CSF and plasma biomarkers in PD has been of prime importance for the diagnosis of disease pathology or the associated neuropathological change in an early stage [178,179]. The study on astrocytic and oligodendrocyte derived exosomes from plasma of PD patients showed a high increase in early-stage PD patients and the concentration of exosomes corroborated with the disease severity [180]. Another study performed where the protein profiling of the plasma-derived exosomes from PD patients at Hoehn and Yahr (HY) stages one, two, and three was done suggested apolipoprotein A1 can be a potential biomarker to monitor disease progression of PD [181]. The level of the α-syn oligomer, α-syn oligomer/α-syn total in salivary exosomes were found to be higher in PD patients but did not correlate with the disease severity [182]. The level of protein DJ1 was observed to be higher in PD patients [183]. Some key studies elaborating on the role of exosomes in the early diagnosis of neurodegenerative diseases are tabulated in Table 3.

### 4.3. Other Neurodegenerative Disorders

The two additional, but less prevalent neurodegenerative diseases are Huntington’s disease and amyotrophic lateral sclerosis. HD is linked primarily to the expanded trinucleotide repeat in the huntingtin gene (HTT) which is the pathological carrier-a mutant form of the multi-functional protein huntingtin. ALS is linked to the copper-zinc superoxide dismutase 1 (SOD1) gene mutation and protein inclusions are ubiquitinated and enriched in tar DNA binding protein-43 (TDP-43) and has concomitant behavioral symptoms like FTD. Huntington is another category of prion-based neurodegenerative disease which is autosomal dominant. It is known to panoply the motor, cognitive and behavioral defects. The abnormal amplification of CAG repeats in the Huntington gene is the accountable cause of this disease [222,223]. The mechanism which regulates the production of mutant huntingtin protein (mHTT) still appears translucent. The prevalence rate of HD is 10.6–13.7 individuals per 100,000 [224,225]. The transmission of mHTT between neurons occurs by tunneling nanotubes and/or vesicle mechanism [189]. There are not many studies that are suggestive of the role of exosomes in the transmission of mHTT. A study on 239T cells, overexpressing HTT-exon1polyQ-GFP reports the presence of polyQGFP and amplified duplicate RNA in EVs. The uptake of EVs by neurons is also observed with an increased presence of polyQ-GFP RNA, although it does not result in further toxicity [189,226]. Another study performed on plasma-derived exosomes of HD patients revealed the presence of 13 miRNAs, which are significantly upregulated [216]. The therapeutic role of exosomes from astrocytic stem cells (ASC-exo) in the in vitro model of HD, the result shows decreased production of the aggregate of mHTT of HD [227]. The presence of mHTT is not seen in astrocytes, although it was observed the increased exosome secretion from astrocytes in HD140Q knock-in (KI) mice. The N-terminal mHTT accumulates in the nuclei and forms aggregates, resulting in the secretion of exosomes from cultured astrocytes [228]. The level of total huntingtin protein in the saliva is higher in HD patients when compared to healthy cohorts [229]. 

Amyotrophic lateral sclerosis comes under the umbrella of prion-based neurodegenerative disorders [230]. ALS is a motor neuron disease that focally spreads in lower and upper motor neurons. It spreads in the motor neuron of the motor cortex, brainstem, and spinal cord [231]. The progression of this disease varies from 3-5 years to extreme slow progression in some affected individuals [231]. The disease shows diverse symptoms ranging from early-onset in the spinal cord characterized by muscle weakness in lower limbs to the bulbar-onset characterized by dysarthria and dysphagia [232]. The impairment in cognition is an accomplice to the cardinal symptoms of ALS [232]. The global prevalence rates of ALS are 5 affected people amongst 100,000 [231]. The most significant genetic link of ALS has persisting connections with the SOD1 gene, which encodes the superoxide-dismutase protein [233]. SOD1 is responsible for the conversion of super-oxide molecules to hydrogen peroxide. There are more than 30 mutations on different genes present amongst which only 14 mutations give rise to the casualty in disease [234,235,236,237,238]. These mutations are involved in protein control, cytoskeletal dynamics, RNA processing, and metabolism, and proteostasis. The diverse mutations recognized in ALS are similar to those accountable for symptoms development in FTD. The presence of C9orf72, an aberrant hexanucleotide (GGGGCC) expansion in the non-coding regions of ALS patients is in similarity to the patients of FTD which characterizes ALS as a neurodegenerative disorder [237,239]. The role of extracellular vesicles in the transmission of ALS pathology in a prion-like manner starts with the accumulation and transport of mutant proteins onto EVs which further aids in transmission across the brain cells [230]. The experimental observations have shown the reduced interaction of C9orf72 and Rab7L1 which are accountable for regulation of a fusion of MVE’s with the plasma membrane [240,241]. SOD1 was the first ALS-associated protein found in EV’s of stable mouse motor neuron-like cells having overexpressed human wild-type and mutant SOD1 [242]. TDP-43, which is known to be the pathological hallmark of ALS, is identified in the brain-tissue isolated exosomes from ALS patients [243]. The secretion of exosomes is also shown to be inhibited with the knocked-out GW4869 OR RAB27A gene, responsible for TDP-43 aggregation [244].

## 5. Potential of Salivary Exosomes Based Biomarkers for Early Diagnosis of Neurodegenerative Diseases

**Figure 5:**
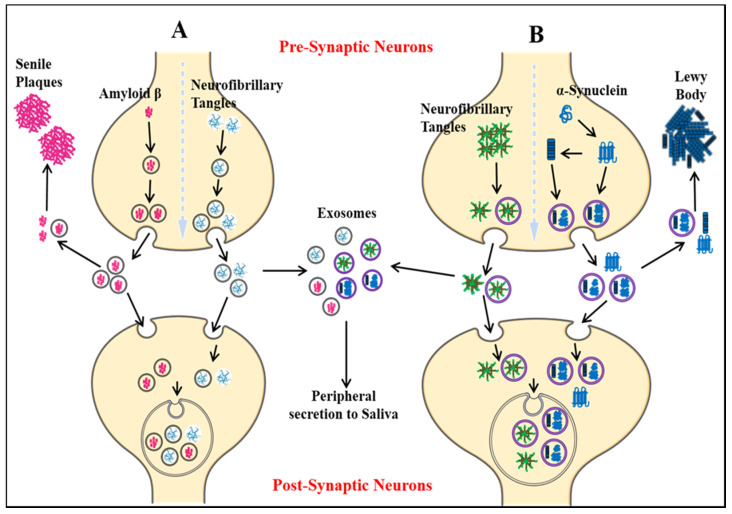
Seeding of hallmark proteins in a prion-based manner by exosomes (**A**) the seeding of amyloid-beta and tau protein through exosomal cargo in Alzheimer’s disease (AD), dispersion of AD pathology. (**B**) the seeding of alpha-synuclein and tau protein in Parkinson’s disease (PD) through exosomal cargo, dispersion of PD pathology. The presence of all these prime proteins in the biofluid saliva after the exosomes cross the blood–brain barrier (BBB).

Salivary exosomes have recently occupied the center stage of biomarker research. The potential of salivary exosomal cargo content as biomarker molecules have gained enormous importance in a very short time. Their role in systemic diseases, cancer, and neurodegeneration is a widely exploring field [245,246]. The inclination towards the usage of salivary exosomes rather than saliva has its advantages. The usage of the whole saliva had numerous problems that can be circumvented by specific isolation of salivary exosomes instead. The prime reasons include the presence of contaminating elements and a higher abundance of amylase enzyme in the whole saliva that tends to mask target biomolecules [32]. Several key studies showing salivary biomarkers in neurodegenerative diseases are tabulated in Table 4. Firstly, the presence of exosome-like “vesicles” in saliva was observed in a study, where an exosome-like structure was observed in electron microscopy after isolation and gel-filtration on Sepharose CL-4B [247]. Salivary exosomes have become a topic of prime importance over the CSF and blood-based biomarkers due to the ease of its isolation from the whole saliva and with no interference from the high abundance protein as in the case of serum. Moreover, saliva enjoys the benefit of being non-invasive in comparison to CSF and blood-based biomarkers [248]. The easy accessibility of saliva enables a potential source for exosomal biomarkers for diagnostic and prognostic assessments (Figure 5).

There are two significant studies on salivary exosomes in Parkinson’s disease. One amongst them reported the content of α-synOlig and α-synOlig/α-synTotal is high in extracellular vesicles of salivary origin in PD patients than healthy control [182]. Another study reported a higher concentration of neuronal salivary exosomes in PD patients in comparison to healthy controls as well as the L1CAM and α-syn protein abundance was also high in PD patients [204]. The only reported study connecting cognitive impairment and Alzheimer’s disease showed a high concentration of salivary exosomes in CI and AD than healthy controls. The amyloid-beta oligomer and p-tau showed high protein abundance in CI and AD in comparison to control subjects [184]. Though these initial studies are exciting for this newly evolving field of salivary exosomes in early diagnosis of neurodegenerative diseases, there is an urgent need for validation studies in terms of the inclusion of a higher number of patients that are monitored for a longer period.

## 6. Concluding Remarks

The degenerative disorders of the nervous systems are known to cause chronic brain-dysfunction. The major neurodegenerative disorders are caused by the aggregation of misfolded proteins and have a multitude of genetic mutations associated with each one of them. The four major neurodegenerative diseases are AD, PD, HD, and ALS. To minimize the burden of these diseases there is a dire need for biomarkers, which can be used to screen these diseases early and in a non-invasive way. Exosomes are nano-sized extracellular vesicles, which are in high abundance within biofluids and have a tendency to cross the blood–brain barrier (BBB). Recently, the role of exosomes is associated with the seeding of these disease pathologies through a prion-based manner. Since these vesicles are able to cross BBB, they can give a fine imprint of the CNS associated disease pathologies. The richness of the peripheral system in these vesicles and their secretion from all cell-types makes them a potent biomarker candidate in many diseases including neurodegenerative diseases. Numerous studies have examined the association of CSF and blood-derived exosomes with neurodegenerative diseases. The usage of another non-invasive biofluid, saliva, and the vesicles derived from saliva has recently gained prime importance as potent biomarker candidates. Here, in this article, we have elucidated the emerging role of salivary exosomal biomarkers their isolation methodologies, and characterization techniques in neurodegenerative diseases. A brief description of the disease pathologies and the role of extracellular vesicles in each of these neurodegenerative disorders is also explained. In the foreseeable future, we hope this current work will serve as a primer for any further studies targeting this emerging area of salivary exosomes as a promising biomarker for various neurodegenerative diseases.

## Figures and Tables

**Table 1:** Key difference between exosomes and microvesicles.

	Exosomes	Microvesicles
Size	30–150 nm	50–1000 nm
Morphology	Cup-shaped	Heterogeneous
Density	1.1–1.2 g/mL	1.08–1.19 g/mL
Origin	Multivesicular Endosomes (MVEs)	Plasma Membrane
Contents	Protein, miRNA, mRNA	Protein, miRNA, mRNA
Protein markers	Alix, Tsg101, CD 81, CD 82, CD63, CD 37, CD9	Selectins, Integrins, CD40

**Table 2:** Techiniques for characterization of exosomes.

Technique	Principle	Advantages	Disadvantages
Electron Microscopy (EM)	Electron radiation	Direct imaging of exosomes, higher resolution, ultrastructure, surface topography	Expensive, cumbersome processing and preparation, qualitative, low throughput
Atomic Force Microscopy (AFM)	Hooke’s law	Higher resolution, sample processing, surface topography, and substructure	Expensive, Low throughput, qualitative
Nanoparticle Tracking Analysis (NTA)	Brownian motion and Stokes–Einstein equation	Size and concentration simultaneously	Better for smaller particles
Dynamic Light Scattering (DLS)	Brownian motion	Simple and fast	Not for heterogeneous populations, lower resolution
Tunable Resistive Pulse Sensing (TRPS)	Coulter principle	Size, concentration, and zeta potential simultaneously	Better for larger particles, cannot differentiate between exosomes and other particles
Western Blotting and ELISA	Antigen-antibody interactions(immunoaffinity)	Detection of exosome-specific proteins, size, and abundance of proteins	Low specificity and quality, higher cost, cross-reactivity
qRT-PCR	Amplification using primers and PCR	Quantitative, low sample volume, higher resolution, high throughput	Limited to the analysis of known target RNA sequences
Flow Cytometry	Coulter principle, light scattering, fluorescence tags/antibodies	Sample processing, fast, specific, reproducible, quantitative, low sample volume	Size standards do not correlate correctly, limitation on detection of lower sized particles

**Table 3:** Studies describing the potential role of exosomes as an early diagnostics for neurodegenerative diseases. (“__” signifies no studies are reported in this subcategory).

Neurodegenerative Disease	Source of Exosome	Studied Exosome Cargo Content	References
Alzheimer’s disease	Saliva	- Aβ-oligomer, p-tau	[184]
	CSF	- Aβ(1-42), total-tau, pT181 - HSPA1A, NPEPPS, PTGFRN - miR-16-5p, miR-125b-5p, miR-451a, miR-605-5p, miR-125-5p, miR-605-5p, miRNA-193b	[185,186,187,188,189,190,191,192]
	Plasma	- βsite amyloid precursor protein cleaving enzyme (BACE-1), γ-secretase - soluble Aβ-42, soluble APPβ, soluble APP α, TDP-43, GDNF, p-T181 tau, and p S396 tau - REST, HSF-1, Lamp 1 and IRS - Synaptic proteins: Synaptophysin, synaptopodin, synaptotagmin-2, and neurogranin - Autolysosomal proteins - Complement proteins: C1q and C4b, Factor D, Fragment Bb, C5b, C3b, C5b-C9 - miR-212, miR-132, hsa-miR-451a, has-miR-215-3p, has-miR-23a-3p, has-miR-216-3p, has-let-7i-5p, has-miR-151a-3p - Insulin receptor substrate (IRS) - P-Serine-312-IRS-1 and P-pan-tyrosine-IRS-1	[135,193,194,195,196,197,198,199,200]
	Serum	- miR-135a, miR-193b, and miR-384	[201]
	Urine	- Aβ (1-42), pS396-tau - Annexin2 and Clusterin	[202,203]
Parkinson’s disease	Saliva	- α-synOlig and α-synOlig/α-synTotal - L1CAM and α-syn protein abundance	[182,204]
	CSF	- α-synuclein, DJ-1 - miR-1, miR-19b-3p, miR-153, miR-409-3p, miR-10a-5p, let-7g-3p, miR-485-5p - miR-433, miR-136-3p, miR-370, miR-873-3p - pS1292-LRRK2	[128,205,206]
	Plasma	- α-synuclein, DJ-1, tau - lnc-RNA-POU3F3	[180,207,208,209,210,211]
	Urine	- pS1292-LRRK2 - LRRK2 14-3-3	[212,213]
Huntington Disease	Saliva	___	___
	CSF	- miR-135b-3p, miR-140-5p, miR-520f-3p, miR-3928-5p, miR-4317, and miR-8082	[214]
	Plasma	- mHtt - miR-877-5p, miR-223-3p, miR-30d-5p, miR-128, miR-22-5p, miR-223-5p, miR-222-3p, miR-338-3p, miR-130b-3p, miR-682-3p, miR-361-5p, miR-425-5p	[189,215,216,217]
	Urine	____	___
Amyotrophic Lateral Sclerosis	Saliva	____	___
	CSF	- TDP-43 - miR-132-5p, miR-132-3p, miR-143-3p - CUEDC2	[218,219,220]
	Plasma	- miR-183-5p - miR 27a-3p - miR-146a-5p, miR-151a-5p, miR-199a-3p, miR-199a-5p - FUS	[218,221]
	Urine	____	____

**Table 4:** A detailed list of studies highlighting salivary biomarkers in neurodegenerative diseases.

Disease	Biomarkers	Outcomes	Methods	References
Alzheimer’s disease	Aβ-42	-Increased in saliva Aβ-42 in MCI, AD patients in comparison to HC -No differences in Aβ-42 in PD and healthy.	ELISA, Sandwich Immunoassay on Magnetic Nanoparticles ELISA kits, detection assay is based on the immunoreaction between the target proteins and their corresponding pair of antibodies followed by fluorescence labeling with a newly developed indolium-based turn-on fluorophore, namely SIM	[249,250,251,252,253,254]
	Aβ-42	-The association between saliva Aβ-42 levels and AD was independent - Decrease in Aβ42	ELISA Magnetic Bead Panel—Multiplex Assay kits	[204,250,255]
	Aβ40	-Aβ40 expression was unchanged within the entire studied sample	ELISA,	[250,253,255]
	Complement C4	-Increase in complement C4	Magnetic Bead Panel—Multiplex Assay kits,	[255]
	t-Tau protein, p-Tau/t-Tau (S396), p-Tau/t-Tau ratio, p-tau	-t-Tau expression in AD patients is significantly lower. -(S396) p-tau/t-tau ratio was significantly elevated in patients with CSF in AD -Higher abundance of p-tau in MCI in comparison to AD -Higher protein abundance in AD and MCI in comparison to healthy controls	Western blotting, Immunoprecipitation, Mass spectrometry, Luminex assays,	[136,256,257]
	total tau (t-tau) tau441, and p-tau181	-No difference in salivary t-tau concentration found between AD and MCI or healthy elderly control -No association of salivary t-tau concentration with neurophysiological assessment or structural magnetic resonance imaging -No significant change	Human Total Tau assay Ultrasensitive single-particle molecule array technology ELISA kits, detection assay is based on the immunoreaction between the target proteins and their corresponding pair of antibodies followed by fluorescence labeling with a newly developed indolium-based turn-on fluorophore, namely SIM	[253,258]
	Lactoferrin	-Salivary lactoferrin shows a very high correlation with all MCI and AD patients	MALDI-TOF/TOF mass spectrometer	[259]
	Lactoferrin	-Reduced in patients suffering MCI and sporadic AD -Decreased levels in PD	Meta-analysis, ELISA, Amyloid-PET scan	[137,260]
	Acetylcholinesterase (AChE)	-The activity of the enzyme was significantly lower in people with AD. Significant age-related decrease in the enzyme	Ellman colorimetric method	[261,262]
	Acetylcholinesterase (AChE)	-No statistically significant -Activity of AChE and PChE significantly increased in the group with AD	Ellman colorimetric method	[138,263]
	Sphinganine-1-phosphate, ornithine, phenyllactic acid, alpha-amyloid protein	-Sphinganine-1-phosphate, ornithine, phenyllactic acid, inosine, 3-dehydrocarnitine, hypoxanthine in the saliva, of the AD subjects were significantly different from the control sphinganine-1-phosphate, which was upregulated in AD	Metabolomics, faster ultra-performance liquid chromatography (FUPLC) mass spectrometry (MS)	[264]
	Higher metabolites level may distinguish AD from CN and MCI with good diagnostic ability	-**Increased**—Methylguanosine, Histidinyl-Phenylalanine, Choline-cytidine, Glucosylgalactosyl Hydroxylysine, Glutamine-carnitines	Metabolomics, liquid chromatography, mass spectrometry	[265]
	Different markers	-**Increased**—Imidazole, Acetone, Creatine, 5-Aminopentanoate, Propionate, and Acetone -**Decreased**—Galactose. Altered metabolites may predict early AD and MCI	1-H NMR	[266]
	Exosomal Aβ oligomer and p –tau	-**Increased**—Aβ oligomer abundance and phospho-tau in AD and CI	Fluoroscence-NTA, western blotting, TEM	[184]
**Parkinson’s disease**	α-syn levels, a ratio of phospho α-syn/total α-syn, total α-syn, α-syn (Oligo and total) a-syn total/oligo α-syn and the extinction coefficient of the saliva protein α-syn (CSF, Plasma, Saliva)	-Increased α-synolig, α-synolig/α-syntotal in PD significantly higher -The α-synolig/α-syntotal ratio was also higher in patients Increase in α-synolig level and α-synolig/α-syn total ratio and a decrease in α-syntotal level among PD patients -α-syn level is significantly less in PD Total saliva protein and uncontaminated protein with nucleic acids are significantly higher in PD -In CSF, α-syn was lower in PD but Plasma and saliva α-syn did not differ between PD and controls	Unstimulated whole saliva, XYCQ EV enrichment Kit, Western blotting, Luminex multiplex assays ELISA, Western blotting, NTA, Sandwich ELISA BioFIND, ELISA	[182,204,210,267,268,269,270,271]
	α-syn (CSF, Plasma, Saliva); total α-syn, oligo α-syn and α-syn SNP variants levels	-Plasma and saliva α-syn do not differ between PD and controls. Saliva α-syn neither correlate with CSF α-syn nor distinguish PD from controls	BioFIND, ELISA	[272]
		-No difference in salivary total α-syn levels was found between PD patients and HC, it decreased with age in PD patients and was closely associated with the genotypic distribution of rs11931074 and rs894278 in PD	Luminex assay, Gel filtration chromatography and Western blot; PCR and sequencing	[273]
	DJ-1	-DJ-1 levels tended to increase in Parkinson’s disease, DJ-1 levels correlated with disease severity	Unstimulated whole saliva, Western blotting, Luminex multiplex assays	[183,213,271]
	DJ-1	-DJ-1 levels in the saliva were not changed significantly in PD patients -A significant difference in salivary DJ-1 levels in PD patients based on different disease stages and clinical subtypes -Salivary DJ-1 levels in the advanced stage of PD were significantly higher than those in the early stage of PD	Luminex assay	[274],
	Acetylcholinesterase (AChE)	-Increased salivary AChE activity -AChE activity/total protein ratio was significantly increased in PD patients	colorimetric method	[275]
	Heme oxygenase-1	-Significantly higher mean salivary heme oxygenase-1 concentrations in patients with H and Y stage 1 PD (early) than control subjects and stage 2 and stage 3 PD patients	ELISA, Western Blotting	[276]
	miRNA-153, mi-RNA-223, miR-7a and miR-7b, mi-RNA 874, mi-RNA 145-3p	-mi-RNA levels decreased in PD patients but did not correlate to disease progression. miR-7a and miR-7b did not correlate with PD or patients. -Ratio of oligomeric α-syn/miR-153 was significantly increased among PD patients, ratio of oligomeric α-syn/miR-223 was not significantly different in the PD patients	RT-qPCR, ELISA	[277,278]
	Exosomal L1CAM and α-syn protein abundance	-Increased α-syn protein in PD patient	NTA, Western blotting	[204]
	α-synOlig and α-synOlig/α-synTotal	-α-synOlig and α-synOlig/α-synTotal significantly higher in PD patients than healthy controls	NTA, Western blotting	
**Healthy**	MicroRNA, Piwi-Interacting RNA, and Circular RNA	-miR-223–3p, miR-148a-3p	Unstimulated whole saliva, Bioinformatics analysis	[279]
**Huntington disease**	Interleukin-6	-The increased level of IL-6 in HD patients in comparison to healthy controls	ELISA	[280]
**Amyotropic lateral Sclerosis**	Chromogranin A	-Higher in terminal ALS patient in comparison to moderately suffering ALS patient	YK070 chromogranin A EIA kit	[281]
		-Correlation of Raman data of ALS patient’s saliva revealed a direct	Raman Spectroscopy	[282]
